# *QuickStats:* Percentage* of Adults Aged ≥18 Years Who Had an Unmet Mental Health Care Need Because of Cost in the Past 12 Months,^†^ by Age Group and Sex — National Health Interview Survey, United States 2019^§^

**DOI:** 10.15585/mmwr.mm6943a8

**Published:** 2020-10-30

**Figure:**
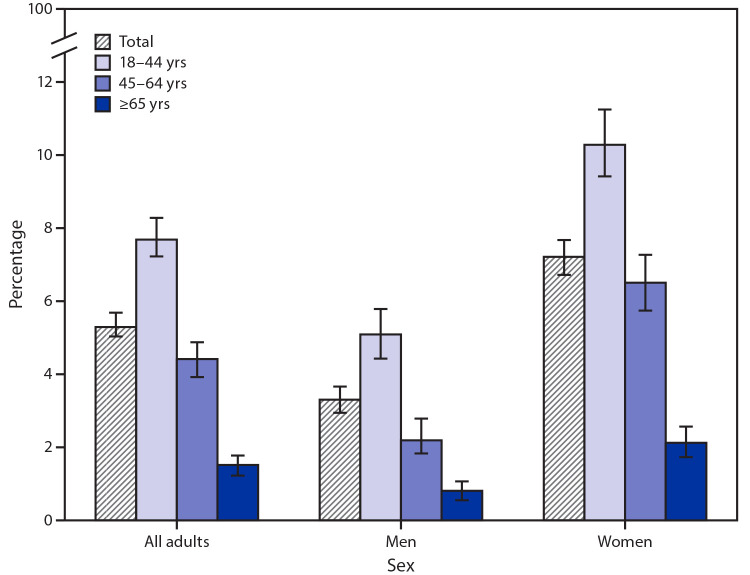
In 2019, 5.3% of adults aged ≥18 years had an unmet mental health care need because of cost in the past 12 months. Women (7.2%) were more likely than men (3.3%) to have an unmet mental health care need because of cost, regardless of age group. The percentage of men with an unmet mental health care need decreased with age, from 5.1% among those aged 18–44 years to 0.8% among those aged ≥65 years. Similarly, the percentage among women decreased with age, from 10.3% among those aged 18–44 years to 2.1% among those aged ≥65 years.

**Source:** National Center for Health Statistics, National Health Interview Survey, 2019. https://www.cdc.gov/nchs/nhis.htm.

